# An eQTL in the *cystathionine beta synthase* gene is linked to osteoporosis in laying hens

**DOI:** 10.1186/s12711-020-00532-y

**Published:** 2020-02-24

**Authors:** Dirk-Jan De Koning, Nazaret Dominguez-Gasca, Robert H. Fleming, Andrew Gill, Dominic Kurian, Andrew Law, Heather A. McCormack, David Morrice, Estefania Sanchez-Rodriguez, Alejandro B. Rodriguez-Navarro, Rudolf Preisinger, Matthias Schmutz, Veronica Šmídová, Frances Turner, Peter W. Wilson, Rongyan Zhou, Ian C. Dunn

**Affiliations:** 1 Swedish University of Agricultural Sciences, 75651 Uppsala, Sweden; 2 Departamento de Mineralogía y Petrologia, Universidad de Granada, 18002 Granada, Spain; 3 The Roslin Institute, University of Edinburgh, Edinburgh, EH25 9RG Scotland, UK; 4 Lohmann Tierzucht, 7454 Cuxhaven, Germany; 5 Present Address: Hebei Agricultural University, Baoding, 071001 Hebei China; 6 Present Address: Department of Chemistry and Biochemistry, Mendel University in Brno, Zemedelska 1, CZ-613 00 Brno, Czech Republic; 7 Present Address: School of Chemistry, The University of Lincoln, Lincoln, LN6 7TS England, UK

## Abstract

**Background:**

Skeletal damage is a challenge for laying hens because the physiological adaptations required for egg laying make them susceptible to osteoporosis. Previously, we showed that genetic factors explain 40% of the variation in end of lay bone quality and we detected a quantitative trait locus (QTL) of large effect on chicken chromosome 1. The aim of this study was to combine data from the commercial founder White Leghorn population and the F2 mapping population to fine-map this QTL and understand its function in terms of gene expression and physiology.

**Results:**

Several single nucleotide polymorphisms on chromosome 1 between 104 and 110 Mb (galGal6) had highly significant associations with tibial breaking strength. The alternative genotypes of markers of large effect that flanked the region had tibial breaking strengths of 200.4 vs. 218.1 Newton (P < 0.002) and, in a subsequent founder generation, the higher breaking strength genotype was again associated with higher breaking strength. In a subsequent generation, cortical bone density and volume were increased in individuals with the better bone genotype but with significantly reduced medullary bone quality. The effects on cortical bone density were confirmed in a further generation and was accompanied by increased mineral maturity of the cortical bone as measured by infrared spectrometry and there was evidence of better collagen cross-linking in the cortical bone. Comparing the transcriptome of the tibia from individuals with good or poor bone quality genotypes indicated four differentially-expressed genes at the locus, one gene, *cystathionine beta synthase* (*CBS*), having a nine-fold higher expression in the genotype for low bone quality. The mechanism was *cis*-acting and although there was an amino-acid difference in the CBS protein between the genotypes, there was no difference in the activity of the enzyme. Plasma homocysteine concentration, the substrate of CBS, was higher in the poor bone quality genotype.

**Conclusions:**

Validated markers that predict bone strength have been defined for selective breeding and a gene was identified that may suggest alternative ways to improve bone health in addition to genetic selection. The identification of how genetic variants affect different aspects of bone turnover shows potential for translational medicine.

## Background

Bone fractures and other forms of skeletal damage are a challenge for laying hens [1] and are the result, at least in part, of progressive osteoporosis [2]. Osteoporosis in hens is ultimately the result of the physiological changes that occur because of the start of reproductive activity. At this stage, the hen starts to form medullary bone [3], which is a specialised bone formed as an adaptation for laying a calcareous cleidoic egg. Medullary bone provides a reserve of calcium for mineralisation of the eggshell and it is very labile, turning over rapidly with the daily cycle of egg laying [3]. This rapid turnover is characterised, as in the structural cortical bone, by osteoblastic and osteoclastic remodelling [4, 5], but there is a rapid change in the rate of mineralisation that depends on the stage of shell calcification. Osteoblast activity in structural cortical bone at this time is minimal, since resources transfer to the medullary bone while osteoclast bone resorption continues. Overall, this is thought to lead to a reduction in the integrity and mass of the structural bone over the period of laying, which can be exacerbated by any imbalance in calcium supply from the diet [6]. These factors are in turn predictive of breaking strength [7], which in turn are predictive of likelihood of fracture or deformations [8, 9].

Whereas undoubtedly housing and nutrition must be optimised to ensure good bone health in laying hens, we believe that genetics offers an important route to reduce bone fractures [10], especially with the increased challenges to hen welfare posed by alternative housing [1]. We have shown that genetics has a clear potential to improve bone health without detriment to production traits: in our previous work, we found that genetic factors underlie the variation in the susceptibility of individual birds to osteoporosis and bone fracture [8]. Some studies have described quantitative trait loci (QTL) for bone quality in chickens related to osteoporosis, usually from crosses of radically different breeds in which body mass can be an issue [11–13], but few have looked within a commercially relevant layer population as investigated here. Divergent selection from a commercial pedigree founder breed on the basis of a bone index (BI), which comprises several bone strength and other traits, resulted in the production of high (Hi) and low (Lo) bone strength lines of laying hens, but with no change in body weight [8]. Selection resulted in the Hi line showing an improvement in tibia strength of over 50%, without any adverse effect on egg production or egg quality. The bones of the hens from the Hi bone strength line have fewer osteoclasts, and consequently suffer less bone resorption during the laying period, resulting in a lower rate of endosteal cortical bone loss and greater accumulation of medullary bone than those from the Lo line [10]. In these hens, there were differences in the degree of collagen cross-linking [14]. Pyrrolic cross-link content of collagen, known to be correlated with osteoporosis in hens, was higher in the humerus and tibiotarsus of the Hi line selected hens [15]. Using an F2 population created from the Hi and Lo lines, a QTL of large effect was characterised on chicken chromosome 1 [16]. In the work reported here, we have fine-mapped this QTL and combined the data with next-generation sequencing of RNA from the bones of hens segregating for markers of the QTL. Ultimately, the genetic markers identified can be used to select for better bone strength, which will reduce the propensity for osteoporosis and, in turn, bone breakage. Few of the many QTL detected in GWAS studies for human osteoporosis have been functionally characterised [17]. Understanding the potential underlying causes that can be deduced from the identification of the genes involved in this QTL may lead to understanding the causes of osteoporosis, suggest new management or nutritional solutions for hens, and potentially lead to a better understanding of bone loss in other species.

## Methods

### Populations of White Leghorn chickens used to fine-map the QTL for bone strength and understand its function

#### Population 1

**Fig. 1:**
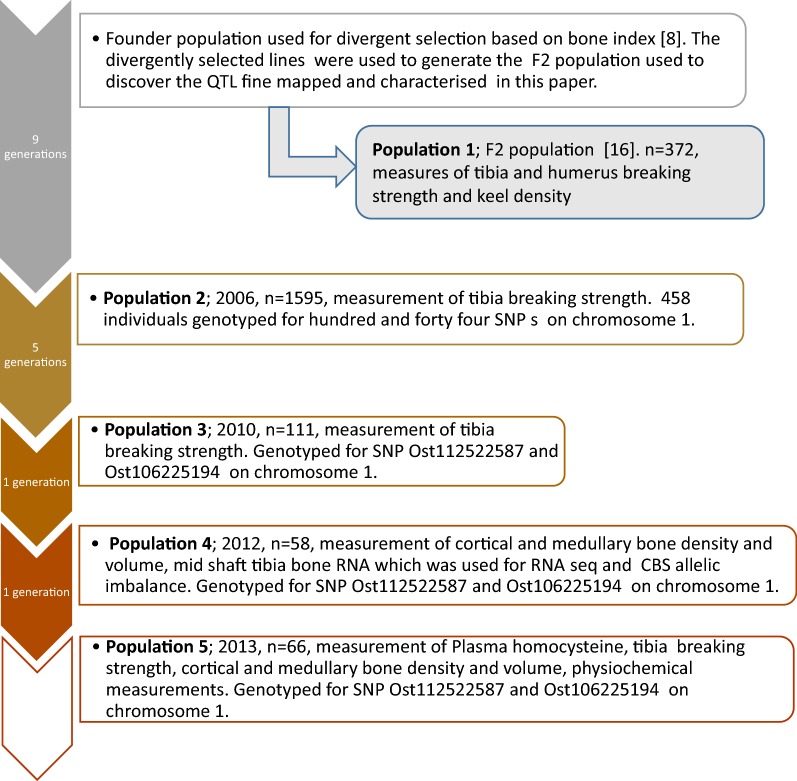
Schematic diagram of the populations used for fine mapping and characterising a QTL for bone quality on chromosome 1. Numbers of generations between the populations are indicated to the left and the year, number of animals, phenotypes and genotypes are in the text boxes to the right

Population 1 was an F2 population (n = 372) that was described previously [16]. It was created by crossing high and low bone quality lines produced by divergent selection of the founder breed [8, 16] (Fig. 1). The QTL on chromosome 1 was originally discovered in this population. The population was used to improve the precision of the location of the QTL with informative single nucleotide polymorphisms (SNPs) at the locus. The measured phenotype was tibial breaking strength and genotyping was performed with microsatellite markers as described previously [16] and SNPs that are listed in Additional file 1: Table S1.

#### Population 2

Population 2 was a later generation (sampled in 2006, see Fig. 1) of the founder breed used to generate the divergently selected population that was used to create the F2 population where the QTL was discovered [16]. It is a White Leghorn breed used in the production of LSL hybrid layers (Lohmann Tierzucht GmbH). With the ability to obtain high-density genotyping and a larger number of animals, the population allowed better precision for mapping the QTL. As in all the populations for which bones were measured in this study, hens were housed in individual cages to facilitate egg recording. Phenotype for tibia breaking strength, body weight and egg production were available for 1595 hens. DNA was prepared from red blood cells using DNAzol (Invitrogen). Hens were genotyped for 144 SNPs as described in the section on genetic markers below and in Additional file 2: Table S2. To increase power and reduce cost only the top and tail of the population were genotyped. Bone strength is influenced by body weight in hens, as witnessed by its negative weighting in the bone index used to divergently select hens that formed the founder population for the QTL analysis [8]. For this reason and to avoid biasing the sample towards heavier and lighter hens, we chose to select equal numbers of hens from the top and tail of the distribution of the population after ranking the hens using the residuals generated after fitting body weight using regression analysis. The equation derived from the data was tibia breaking strength (N) = 53.0 + 0.0946 body weight (g). Hens laying less than 230 eggs were removed, since these can have stronger bones and do not form part of the normal distribution, as was done in the original QTL study [16]. Equal numbers of individuals from the top and the tail of the population were selected and 998 animals were genotyped. This represented 63% of the population. The phenotype was tibial breaking strength and genotyping was performed with 144 SNPs on chromosome 1.

Further studies were undertaken and samples were collected from the founder breed in subsequent years to characterise the locus and its effects.

#### Population 3

Samples (n = 111) for genotyping and phenotyping were taken from the 2010 generation (Fig. 1) of the founder breed using a targeted sampling approach, which was used to provide further independent confirmation of the locus effect. As in the other populations, only hens laying more than 230 eggs were analysed. To obtain the best power with the resources available, samples were collected only from hens at post-mortem if, after being tested for breaking strength, the bones were more than 1.5 standard deviations from their predicted breaking strength (PBS). PBS was calculated using a training set of 200 hens. The equation was produced by regressing tibial breaking strength against body weight and total egg production. (PBS = 90.5 + (− 0.687 egg production) + (0.1239 bodyweight)). The distribution of the ‘residual’ between the observed and predicted breaking strength based on the 200-hen training set was used to define 1.5 standard deviations. This ensured that we did not bias the results since larger hens have stronger bones and hens that lay significantly fewer eggs due to pauses in production can have stronger bones as do out-of-lay hens [18]. Hens with four or less follicles or with any evidence of reproduction abnormality such as internal ovulations were rejected at post-mortem. The phenotype was tibial breaking strength and genotyping was performed with the Ost106225194 and Ost112522587 SNPs.

#### Population 4

Samples of mid-shaft tibia bone were collected from the 2012 generation (Fig. 1) for the preparation of RNA for next-generation sequencing (NGS) and to make bone density measurements. This allowed the characterisation of expression differences related to the QTL. Thirteen sires and 50 dams were genotyped for SNPs Ost106225194 and Ost112522587 and offspring from heterozygous individuals were prioritised for the detection of homozygotes by genotyping the same markers. Homozygous hens (n = 34 *AA*/*AA*; n = 22 *GG*/*GG*) were phenotyped at the end of their productive cycle at 70 weeks of age. RNA was prepared from mid-shaft tibia. The measured phenotype was mid-shaft cross-sections of tibia that were taken for radiographic measurement of cortical and medullary bone density, volume and area. The animals used for NGS of bone RNA were selected from these (see section on NGS). Genotyping was performed with Ost106225194 and Ost112522587 SNPs.

#### Population 5

Samples were obtained from the 2013 generation (n = 66) using the same sampling strategy as for Population 4, but samples were collected for evaluating specific properties of bone material at 70 weeks of age to understand how the genotype might result in the observed bone strength phenotype (Fig. 1). The measured phenotype was concentration of homocysteine in the plasma towards the end of peak egg production (48 weeks of age) and end of production (70 weeks of age). A subset of these animals, n = 19 per genotype, with matching physiology with regard to stage of egg formation were further analysed for bone mechanical properties, bone microstructure, chemical composition of the cortical and medullary bone, mineral crystallinity and crystal orientation and collagen maturity using infrared spectroscopy and X-ray diffraction techniques. Genotyping was performed with Ost106225194 and Ost112522587 SNPs (n = 33 *AA*/*AA*; n = 33 *GG*/*GG*).

#### Population 6

Population 6 was a White Leghorn line that was not related to the other populations. We used it to provide material for the measurement of CBS enzyme activity as described in the respective sub-section. It consisted of embryos from a White Leghorn population maintained at the Roslin Institute, which was found to carry the SNP rs316554658 that resulted in a predicted amino acid difference. Sires and dams heterozygous for this SNP were bred and the resultant eggs were incubated. At day 15, the embryo livers were harvested and stored at -20 °C prior to use in the assay, and a sample of blood was taken for DNA preparation to confirm the genotype of the embryo. The measured phenotype was CBS enzyme kinetics and genotyping was done with SNP rs316554658.

### Genetic markers

For fine mapping, SNPs in the QTL region were sourced in a number of ways. SNPs were sourced from dbSNP [19] along chicken chromosome 1 for use in the F2 cross. A more targeted set of SNPs was defined from sequence information [20] from White Leghorn breeds between 81.1 and 128.8 Mb on chromosome 1 (galGal6). This corresponded to the 95% confidence interval (CI) of the original QTL. The number of SNPs was reduced from 91,455 to 15,761 by using only those segregating in all the sequenced breeds, and finally to 193 using those that had a minor allele frequency (MAF) lower than 0.2 in all three breeds. This list was reduced furthermore by removing SNPs in close proximity to each other and by biasing the density to be higher at the perceived peak and reduced at the tails of the region. Around 25 SNPs failed the test for ability to construct an Illumina Golden Gate assay. The SNP list was supplemented by SNPs that were derived from the alignment of EST sequences from the following genes located in the target region: *RGN*, *EFHC2*, *EGFL6*, *DMD*, *PHEX*, *AGPAT3*, *PRDM15*, *MX1*, *PIGP*, *MRPS6*, *DDX3X*, *RPL8*, *ATP6AP2*, *PDK3* and *APOO*. The full list of genotyped SNPs is in Additional file 2: Table S2. One hundred and forty-four SNPs were genotyped on Population 2 using the Illumina Golden Gate assay on the Illumina BeadXpress platform. The Illumina Bead Studio Genotyping Module software was used to analyse SNP data and, as a first quality control, to remove poor quality or uninformative SNPs. From the 119 SNPs obtained from sequence data, only 13 were informative in the F2 cross, whereas in the case of the SNPs discovered using EST sequences, 19 out of 39 were informative. The 95% CI of the QTL was chr1: 75,624,794-124,047,308 bp (galGal6) and the region covered by the new markers was chr1: 77,312,158-124,272,094 with an average distance of 0.336 Mb (SD 0.183) between SNPs.

For Population 1, the 32 informative SNPs from those detailed above were genotyped using the Illumina Golden Gate assay on the Illumina BeadXpress platform. The Bead Studio Genotyping Module software was used to analyse SNP data. These SNPs were added to the genetic map for chromosome 1 to determine the QTL position in the F2 Population 2. The new F2 map is in Additional file 1: Table S1. The map was used as previously [16] using the QTL mapping method of Haley et al. [21] but implemented using gridQTL [22].

### Small-scale genotyping

Genotyping with SNPs Ost112522587 and Ost106225194 to define parents or individuals for Populations 3, 4 and 5 with the desired genotype was carried out by LGC (LGC, Middlesex, UK), assay reference Chr1_112522587200 and Chr1_1062251946, respectively.

SNP rs316554658, which was predicted to result in an amino-acid change, was diagnosed using RFLP after amplification with primers CBS_NS_F (5′ CGT CTG GTG AAG GGG AAT AA 3′) and CBS_NS_R (5′ TCC CTT TTC AGC TGC TCA GT 3′). This resulted in an amplified product of 596 bp using genomic DNA as target (Chromosome 1, 106,922,247–106,922,842). When digested with the restriction enzyme HpyCH4V, the amplification of allele *C* gives products of 289, 166, 93 and 48 bp (the restriction site at position 289 in the PCR product is part of a codon for Q at position 498 in the CBS protein, therefore *CC*). Digestion of the allele *A* product (part of a codon for K at position 498 in the CBS protein) gives digestion products of 382, 166 and 48 bp. This was used to genotype Population 6 to allow identification of individuals that differed in their CBS amino acid sequence and to collect tissue to assess the function of the enzyme.

### RNA seq

Mid-shaft tibia bone total RNA samples were prepared from 70-week old hens from Population 4 for which the egg was in the shell gland to reduce any effect of the egg calcification cycle. RNA was prepared using the TRIzol reagent according to the manufacturer’s instructions (Invitrogen Ltd., Renfrewshire, Scotland). RNA was treated with DNAse I and purified using an RNeasy Mini Kit following the manufacturer’s instructions (Qiagen, Manchester, England), concentration and quality were checked with a Nanodrop ND-1000 spectrophotometer (Thermo Scientific; Waltham, MA USA). Half of the samples were homozygous for the low bone breaking strength genotype (n = 8) and the other half (n = 8) were homozygous for the high bone breaking strength genotype. Total RNA samples (1 µg) were prepared for mRNA sequencing using the Illumina Truseq RNA Sequencing protocol. Resulting libraries were quality-checked on an Agilent DNA 1000 bioanalyzer (Agilent Technologies, South Queensferry, UK) and then clustered onto a paired end flowcell using the Illumina v3 cluster generation kit at a concentration of 8 pM. One hundred cycle paired-ended sequencing was carried out on the HiSeq 2000 using Illumina v2 Sequencing by Synthesis kits (Illumina, Little Chesterford, UK). An Illumina HiSeq 2000 platform at Edinburgh Genomics generated between 40 and 60 million RNAseq reads per sample (819 million in total). Quality control of the raw data was evaluated using the FastQC package [23] (Babraham bioinformatics, Cambridgeshire, England). Reads were adapter-trimmed using cutadapt version 1.3 [24] with the parameters-q 30-m 50-a AGATCGGAAGAGC. Differential expression of genes or tags was assessed using edgeR version 3.6.8 [25], a package in the bioconductor suite [26] implemented in R [27]. The likelihood that the expression of genes differed between the genotypes was estimated using a general linear model in the edgeR package. Genes with a false discovery rate less than 0.05 were reported as of interest. A fuller version is available in Additional file 3. The data were submitted to the European nucleotide archive (study accession PRJEB6782) and have been used in the annotation of the chicken genome [28].

Examination of genes of interest using the NGS data was performed manually using Tablet v1.17.08.17 [29] to ascertain the location of polymorphisms in each bird and these were annotated to the sequence using Seqbuild (DNASTAR, Inc. Madison, WI). Assembly of short reads to produce the *CBS* mRNA sequence was made using Staden Gap5 v1.2.14-r [30].

### The *CBS* gene

#### Understanding the CBS gene in the chicken: sequence and variant expression

The *CBS* gene is considered to be present in the chicken genome based on a prediction. It has the Ensembl transcript ID ENSGALT00000026110 at position chr1:111,011,700–111,029,467 (galGal6). Another gene shares sequence similarity in some of the exons with *CBS* and is named the *CBS*-*like* gene (*CBSL*); it has the Ensembl transcript ID ENSGALT00000026104 and is located upstream from *CBS* at position chr1:110,980,772–110,999,637 (galGal6). ENSGALT00000026110 and ENSGALT00000026104 are 1836 bp and 1938 bp long, respectively, and the identity over the regions that align is equal to 73.5% (1106/1505) with 14 gaps using ‘Matcher’ in mEMBOSS [31]. The sequences differ sufficiently for defining specific PCR assays to investigate their relative expression.

#### CBS quantitative PCR

*CBS* gene expression was measured using quantitative PCR (qPCR); a standard curve was used to quantify expression of *CBS* and measurements were standardised using the *lamin B receptor* (*LBR*) as reference gene, the expression of which did not differ between genotypes (P = 0.585). The overall methodology for quantification was as previously described in [32, 33].

cDNA for measurement was prepared from mid-shaft tibia samples from the Ost112522587/Ost106225194, *AA*/*AA* (n = 36) and *GG*/*GG* (n = 25) genotypes from Population 4. Because two versions of the gene appear to exist in the chicken genome as stated above, *CBS* and *CBSL*, we measured both to confirm which gene was differentially expressed. In the process, we established that the *CBSL* gene was not highly expressed using the ΔΔct method [34] to estimate the fold difference in expression of the two genes in the same samples. The *CBS* primers for qPCR avoided polymorphisms that were known to be within the locus: CBS-F2a, TTGGGCTGAAGTGTGAACTC; CBS-R2, TCAGGACATCCACCTTCTCC; product length 233 and for CBSlikeF2, GCTCCGGAGTCTAACATTCG; CBSlikeR, ATCACCACCATGTGGACCTT product length 164 bp. Using 16 samples of RNA extracted from mid-shaft tibia bone for NGS, which represented eight samples of each genotype (Ost112522587/Ost106225194, *AA*/*AA* vs. *GG*/*GG*) that were in the same reproductive state, the version of *CBS* represented by ENSGALT00000026110 was expressed on average 815 ± 157-fold higher than *CBSL* when calculated from the quantitative PCR data. For this reason, we confined subsequent measurements to the *CBS* gene.

#### CBS allelic imbalance

Allelic imbalance of *CBS* expression in hens that were heterozygous at the Ost112522587 SNP from Population 4 was determined by amplification of cDNA derived from bone of 10 heterozygous hens using primers CBSgenoF1, GTGGAACGTCAGTGTTCAGG; CBSgenoR1, AAGGCTGAACTTTTCCAGCA followed by digestion with HpyCH4V restriction enzyme. This yields DNA products of 132 and 68 bp for the *A* allele associated with low bone strength or leaves the fragment uncut at 200 bp for the *B* allele associated with high bone quality. Products were run on a 3% agarose gel containing Sybrsafe (Invitrogen, Paisley, Scotland). The intensity of the bands was calculated using ImageJ 1.32 (http://imagej.nih.gov/ij/) on images taken using a G:Box imager (Syngene, Cambridge, UK). The sum of the intensity of the 132-bp and 68-bp band was compared with the 200-bp band intensity expressed as a fraction of the total area under the curve using a paired *t* test.

#### CBS enzyme activity assay in livers of embryos expressing protein from allelic variants

A coding variant was detected at position 498 in *CBS* coding for glutamine (Q) or lysine (K) giving rise to two allozymes. Estimation of the activity of CBS in liver expressing the protein that contains these alternative amino-acids was based on the production of cystathionine in the presence of varying substrates (L-homocysteine 0.1–5 mmol/L, serine constant at 5 mmol/L) and cofactors (S adenosylmethionine 200 µmol/L and pyridoxal phosphate 50 µmol/L [35]. All reagents were obtained from Sigma-Aldrich. In brief, liver samples from day-15 embryos harvested from Population 6, which segregated for the alternative alleles, were homogenized (Ultraturrax, IKA-Werke GmbH & Co. KG, Germany) in an extraction buffer containing protease inhibitor. Protein concentrations of the homogenate supernatant were estimated using a Pierce™ Coomassie blue assay (Thermo Fisher, UK) and 100 µg of protein was included in the assay in a total volume of 50 µl and incubated for 1 h at 37 °C. Finally, 150 µl of acetonitrile (VWR Chemicals Leicestershire, UK) was added to precipitate proteins and the supernatant retained for liquid chromatography–mass spectrometry (LC–MS) measurement, see below. The Michaelis–Menten constant (Km) and the maximum activity (Vmax) were estimated by plotting a double reciprocal plot using the rate of cystathionine production at each homocysteine concentration; 50, 40, 30, 20, 10, 5, 2 and 1 mM.

#### Measurement of homocysteine and cystathionine using LC–MS

LC–MS measurements for homocysteine and cystathionine levels in samples from the CBS enzyme activity assay were performed by a selected reaction monitoring assay on an amaZon ETD IonTrap Mass spectrometer (Bruker Daltonics, GmbH, Bremen, Germany) coupled to an Ultimate HPLC (Dionex) system, for more details (see Additional files 3 and 4). In addition, the method was used to measure plasma homocysteine for validation of the commercial kit as described below.

#### Plasma homocysteine measurement

Plasma homocysteine was measured using the kit HY4036 (Randox laboratories, County Antrim, UK) based on enzymatic conversion of homocysteine to cystathionine by CBS. Plasma was treated prior to measurement with lipoclear (Vetlab supplies, West Sussex, UK) to remove circulating lipids. In humans, the homocysteine assay relies on low endogenous levels of circulating cystathionine to work, but it was not clear if this was true in chickens. To ascertain if concentrations measured by the kit were the same as those measured using LC–MS, a set of samples was measured by both methods. Comparison of the respective measurements resulted in an R-squared value of 92%, which suggested that the biochemical method worked adequately to detect homocysteine in chicken plasma.

### Bone material properties

#### Breaking strength

Among the main morphological and biomechanical properties, tibia breaking strength was determined by a three-point bending test using a material testing machine (JJ Lloyd LRX50, Sussex, UK) as previously described [7].

#### Radiographic density

Cross-sections of the tibia bone were radiographed in a Faxitron 43855D soft X ray apparatus using Kodak MRE-1 high-resolution mammography film in Min-R2 cassettes with a single Min-R intensifying screen. Each exposed plate included a 16-step aluminium wedge, with 0.25-mm increments, for calibration purposes. Exposed films were developed using an automatic processor and then digitised using a Kodak LS-75 film scanner. Measurement of the radiographic density and proportion of medullary and cortical bone in the tibia was made using the software package ImageJ 1.32 (http://rsb.info.nih.gov/ij/). Each tibia bone was automatically delineated from the background and the mean radiographic density (pre-calibrated in mm of aluminium equivalent) of the whole bone was measured. The proportion of medullary and cortical bone type was calculated directly from the X-ray by delineation [36].

#### Bone chemistry and structure

*Bone samples*: Tibia bones were stored in a freezer at − 20 °C until analysed for bone physicochemical material specific properties (e.g., bone microstructure, chemical composition of the cortical and medullary bone, mineral crystallinity and crystal orientation, and collagen maturity) using infrared spectroscopy and X-ray diffraction techniques as fully described in Additional file 3 and briefly below.

*Infrared spectrometry*: The chemical composition of bone tissues (cortical and medullary bone) were analyzed by infrared spectroscopy as previously described [37]. The relative amounts of water, proteins (collagen), lipids, phosphate and carbonate in the bone samples were determined from the peak area of the absorption bands associated with the characteristic molecular groups of each component [38, 39]. In addition, the absolute water, organic matter, carbonate and phosphate contents in bone were determined by thermogravimetry (TGA) in selected samples. For these analyses, about 25 mg of the powdered bone were introduced into a crucible and analysed using a TGA system from METTLER-TOLEDO (mod. TGA/DSC1). A heating rate of 20 °C/min was used for registering the TGA curves.

*X*-*ray diffraction*: Tibiae cortical bone (about 1 × 1 cm) samples cut from the diaphysis were analyzed in transmission mode with a single crystal diffractometer equipped with an area detector (D8 SMART APEX from Bruker) and Mo radiation (50 kV and 30 mA; 0.5 mm collimator). A quantitative estimation of the degree of orientation of apatite crystals (Angular spread; AS) in the cortical bone was determined from the angular breadth of bands displayed in the intensity profile along the Debye–Scherrer ring associated with the 002 reflection of apatite mineral [37].

## Results

### Fine mapping

#### Improved resolution of the QTL in Population 1

To improve the resolution in Population 1, which is the original F2 used to detect the QTL on chromosome 1, 27 new informative markers from those used on Population 2 were added to the original map. The F value for the QTL improved from 13.2 in the original publication to 16.0 for the tibial breaking strength trait, from 7.9 to 10.9 for humeral breaking strength, and from 9.3 to 12.3 for the bone index compound trait. Overall, the position of the QTL became more consistent at 363–365 cM compared to the original estimates and the 95% confidence interval decreased (Table 1).

**Table 1:** Estimates of the position of the bone quality QTL located on chromosome 1 in hens of an F2 reciprocal cross between White Leghorn hens divergently selected for bone index

Trait	Position (cM)	95% CI (cM)	F statistic	Flanking markers	^a^Position (Mb)
Tibiotarsal breaking strength	363 (370)	348–402 (138–416)	16.2 (13.2)	ROS0081 ADL0148	111.2–113.1
Humeral breaking strength	365 (343)	129–381 (30–376)	11.1 (7.9)	ADL0268-MCW0061	90.2–90.2
Bone index	364 (364)	325–401 (198–393)	12.3 (9.3)	Ost109151638-ADL0148	107.0–113.1

The values in brackets represent the values found in the original analysis [16]

^a^Positions in Mb on the chicken genome galGal6 build were calculated from the position of the flanking markers on the 2011 (galGal4) build, then updated using Liftover

**Fig. 2:**
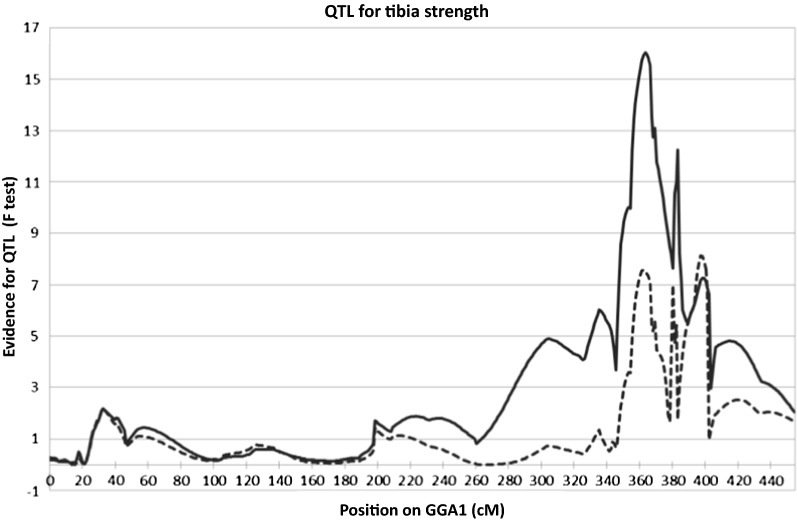
Evidence for a QTL affecting bone strength in an F2 population on chicken chromosome 1 (solid line) and with the most significant SNP from the association study fitted as a covariate (dashed line)

When the most significant SNP from the association study was fitted as a covariate, the QTL effect was in great part removed (Fig. 2).

### Identification of the functional consequences of the QTL

#### Trait values and marker association in Population 2

For Population 2, the 2006 generation of the study population, which was measured at 70 weeks of age, egg production, body weight and tibia breaking strength (mean ± SD) of the population were equal to 240.9 ± 10.9 eggs, 1623 ± 162 g and 206.5 ± 42.1 Newton, respectively (n = 1595).

The results of the association analysis using the 32 SNPs that segregated at the QTL are in Table 2. The average effect size for markers with significant effects is around 15 Newton between the homozygotes with the additive effect representing half this value. The alternative combined genotypes for the most significant SNPs of large effect and flanking the region (Ost112522587/Ost106225194; *AA*/*AA* vs. *GG*/*GG*) had tibial breaking strengths of 200.4 vs. 218.1 Newton (P < 0.002).

**Table 2:** Significance of association between SNPs on chromosome 1 and tibial breaking strength in hens at 70 weeks of age

Marker name	GalGal6 chr 1 position (bp)	F statistic	P value	Heterozygote effect (Newton)	Homozygote effect (Newton)
Ost92365348	90,332,842	0.22	0.802		
Ost99883015	97,883,872	1.6	0.203		
Ost101413556	99,395,470	2.04	0.131		
*Ost106225194*	*104,084,042*	*7.45*	< *0.001*	*6.08*	*16.02*
Ost106460620	104,314,846	2.88	0.057		
Ost106823022	104,678,816	3.85	0.022	12.77	2.62
Ost106940170	104,834,987	4.9	0.008	6.336	17.15
*Ost107389494*	*105,280,127*	*6.3*	*0.002*	*13.2*	− *1.65*
Ost107766125	105,655,660	5.58	0.004	12.61	4.83
Ost108015093	105,901,013	5.59	0.004	12.69	4.82
Ost109151638	107,036,767	3.17	0.042	− 7.75	− 16.75
Ost109151769	107,036,898	3.17	0.042	9.00	16.75
Ost110373245	108,261,209	3.18	0.042	− 0.58	− 8.29
Ost110455031	108,342,396	5.49	0.004	6.59	18.46
Ost112374543	110,246,492	3.51	0.03	− 2.98	− 12.63
*Ost112522587*	*110,393,717*	*6.34*	*0.002*	*9.92*	*16.55*
Ost113740421	111,546,531	1.59	0.205		
Ost113743229	111,549,338	1.29	0.276		
Ost114648871	112,392,632	0.99	0.372		
Ost115611476	113,329,344	3.66	0.026	− 3.8	− 11.13
Ost115617544	113,335,412	0.44	0.647		
Ost115655839	113,373,156	2.47	0.085		
Ost115861595	113,578,876	1.97	0.14		
Ost115862305	113,579,586	1.78	0.17		
Ost115865281	113,582,562	1.4	0.248		
Ost115866264	113,583,545	0.16	0.92		
Ost121757410	119,732,454	0.2	0.821		
Ost121237272	119,214,984	6.19	0.013	− 7.67	
Ost126455926	124,863,293	0.27	0.601		
Ost129219873	126,721,130	5.68	0.004	− 46.2	− 61.66
Ost134239048	131,424,865	0.14	0.872		
Ost134239237	131,425,054	0.14	0.872		

F values were obtained by REML analysis with body weight, hatch, total eggs and eggs in the 3 days before death as fixed effects, and sire and dam as random effects. Effect sizes are quoted relative to the reference homozygote only for the SNPs that had a nominal significance level lower than 0.05. If corrected for multiple testing and taking the identification of seven blocks containing 23 SNPs using Haploview [40] into account, then the significance after Bonferroni correction should be set at 0.003. The SNPs passing this threshold are in italic characters. The additive effect represents half that of the homozygote reference

#### Population 3: identifying the functional consequences

In 2010, the founder population (Population 3) was re-sampled and 111 individual hens were identified that differed in their predicted versus their observed tibial breaking strength by more than 1.5 of a standard deviation in each direction. When subsequently markers for bone quality were identified (Table 2), these individuals were genotyped for two of the markers with a large effect that flanked the region (SNPs Ost112522587 and Ost106225194). Hens segregating for the Ost112522587 genotype displayed a large difference in tibial breaking strength although the high bone strength genotype was poorly represented in the sample; *A*:*A*, 198.4 ± 7.2 Newton, N = 83; *G*:*A*, 225.3 ± 13.4 Newton, N = 26; and *G*:*G*, 261.7 ± 27.1 Newton, N = 2. This difference was significant when fitting body weight as a covariate (P = 0.04) and represents an additive effect of about 32 Newton. There was no significant difference in body weight, egg breaking strength, or egg number between genotypes. The size of the effect may have been inflated by the selection procedure for the top and tail distribution of bone strength.

#### Expression analysis of bone from Population 4

With the identification of predictive markers, it was possible to prioritise families, which were likely to contain offspring that would be homozygous at the markers by genotyping the parents (Population 4). Individuals homozygous at Ost106225194 and Ost112522587 did not differ for body weight or eggshell strength (Table 3). The cortical density and cortical bone volume were greater, although not significantly (P = 0.09 and 0.06, respectively), in the group associated with the high bone strength genotype (*GG*/*GG*). However, the most striking difference was for the medullary bone. The medullary area and volume were smaller, but the medullary bone was denser in the high bone strength genotype (Table 3).

**Table 3:** Summary statistics for the animals segregating at SNPs Ost106225194 and Ost112522587

Genotype at Ost106225194 and Ost112522587	^a^*AA/AA*	^b^*GG/GG*	P value
Egg shell thickness (mm)	0.175 ± 0.012	0.197 ± 0.011	0.23
Body weight (g)	1599 ± 19	1600 ± 28	0.97
Total eggs	301.5 ± 2.7	303.0 ± 3.3	0.62
Cortical bone density (units per mm^3^)	0.045 ± 0.002	0.050 ± 0.002	0.09
% as cortical bone	42.44 ± 0.85	44.46 ± 1.427	0.20
Cortical bone volume (mm^3^)	38.30 ± 1.80	33.27 ± 1.70	0.06
Medullary bone density (units per mm^3^)	0.024 ± 0.001	0.032 ± 0.003	0.009
Medullary bone volume (mm^3^)	42.59 ± 2.10	32.33 ± 1.62	0.001
Medullary area (mm^2^)	16.47 ± 0.83	14.02 ± 0.71	0.044
Total area	2440 ± 65	2311 ± 46	0.155

All the bone measurements were made on the left tibia mid-shaft. The *AA*/*AA* genotype is the genotype associated with lower bone strength. ^a^N = 36; ^b^N = 25

**Fig. 3:**
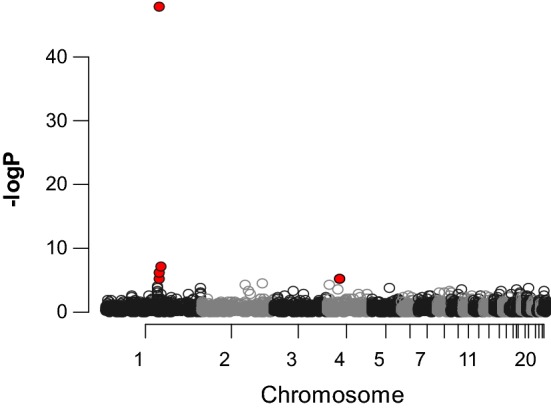
Manhattan plot of the significance (−logP) of the difference in expression of genes in the tibia between hens segregating for markers defining good (n = 8) and poor (n = 8) bone strength. x-axis: chicken chromosomes and distance along the chromosomes. The genes marked in red are those considered significant, the majority are in the region of the mapped QTL on chromosome 1

To understand what expression differences may exist between the genotypes at the QTL, eight hens from each homozygous genotype were selected for transcript profiling by NGS. The hens were a sample of the population represented in Table 3. As in the whole population, there was no difference in body weight or egg production and all hens had an egg in the same position in the shell gland. Analysis of the RNAseq data highlighted five genes that were significantly differentially expressed between the genotypes using a nominal FDR of 0.05 (Table 4). All of these differentially expressed genes mapped to the refined QTL that define good and poor bone strength, with the exception of the gene on chromosome 4, (Fig. 3, and Table 4). In addition, with the exception of the gene on chromosome 4, they were expressed at a higher level in the low bone strength genotype. The most significant transcript, *CBS*, had a P value of around one order of magnitude greater than that of any of the other gene transcripts that passed the significance threshold (Table 4).

**Table 4:** List of genes with significant expression differences in the tibia between hens segregating for markers defining good (n = 8) and poor (n = 8) bone strength

Log fold change	P value	FDR	Ensembl gene ID	Chr	bp	Associated gene symbol
− 1.76	1.50E−48	1.83E−44	ENSGALG00000016196	1	111,011,709	*CBS*
− 0.53	1.02E−07	0.00062	ENSGALG00000022808	1	111,336,046	*RRP1B*
− 0.66	1.00E−06	0.00409	ENSGALG00000016200	1	111,210,236	*SIK1*
1.92	6.64E−06	0.01966	ENSGALG00000009639	4	24,961,239	*DDX60*
− 0.72	8.02E−06	0.01966	ENSGALG00000016048	1	108,339,080	*PIGP*

*FDR* false discovery rate, *Chr* chromosome, *bp* position on the galGal6 build of the starting point of the gene

The differential expression of *CBS* was confirmed (P < 0.001) by qPCR in a larger population of birds including the animals used in the NGS represented in Table 3. The expression of the *CBS* gene was 10.2 ± 1.2 × 10^−12^ (n = 36) in the low bone strength genotype (*AA*/*AA*) vs. 3.4 ± 0.5 × 10^−12^ (n = 25) in the high bone strength genotype (*GG*/*GG*).

#### Allelic imbalance

To reinforce the observation and to demonstrate if the effect was a *cis* or trans effect, expression of each allele in heterozygotes was examined. In heterozygotes from the same generation for which *CBS* expression in bone was measured (Population 4), the relative expression of alleles *AA* and *GG* was significantly different (P < 0.001). The expression of allele *AA* was much higher than that of allele *GG* (0.90 ± 0.01 versus 0.10 ± 0.01; n = 10). This suggested that expression of allele *AA* was ~ 9 times that of allele *GG* within an animal.

#### Physico-chemical measurements of bone, radiographic bone density and plasma homocysteine from Population 5

To probe further into the underlying physiology and chemistry of the effect of the different alleles, we carried out a detailed examination of circulating homocysteine and of bone properties in the alternative genotypes. In a sample from Population 5, hens homozygous for the alternative alleles at the *CBS* gene displayed several statistical differences in bone quality (Table 5). While there was no difference in the gross measures of mechanical bone strength, there was a significantly higher circulating level of homocysteine at 48 and 70 weeks of age in hens with the *AA*/*AA* genotype. The *AA*/*AA* genotype is associated with lower bone quality. Several morphometric and physico-chemical measurements differed significantly in this population. In particular, the cortical bone density, its degree of mineralization (PO_4_/Amide I), cross-linking of collagen LNK 1660/1690, degree of crystal orientation (inversely related to AngSpread002 and crystallinity index CI 1030/1020) were lower, whereas the amount of carbonate in bone mineral was higher in hens with the *AA*/*AA* than in those with the *GG*/*GG* genotype (Table 5). In contrast, there were no effects in the degree of medullary bone mineralization or chemical composition and, as in our previous observations, there was no difference in egg production or eggshell strength between the genotypes (Table 5).

**Table 5:** Values for plasma homocysteine concentration at 48 and 70 weeks of age, production data, radiographic density, mechanical and physicochemical bone properties measured at 70 weeks of age between *CBS* genotype Ost106225194/Ost112522587 (*AA*/*AA* vs. *GG*/*GG*) in Population 5

	Trait	*AA*/*AA* mean	se	*GG*/*GG* mean	se	P value	df
Radiographic, mechanical, metabolite and production data							
Body weight	Body weight (g)	1638	24	1603	24	0.291	1,64
Egg traits	Egg number	282.3	1.8	276.0	2.3	0.574	1,64
	Egg breaking strength (48 wk of age)	40.1	0.9	40.4	1.3	0.508	1,61
Plasma homocysteine	Homocysteine 48 wk of age (µM/L)	18.64	0.55	16.68	0.64	0.024	1,52
	Homocysteine 70 wk of age (µM/L)	19.13	0.46	17.18	0.63	0.016	1,64
Detailed morphometry and physicochemical analysis							
Bone type							
Whole tibia	Breaking strength (N)	228.1	6.6	228.2	9.0	0.998	1,36
Whole tibia	Stiffness (Nm)	316,939	8456	322,940	9526	0.64	1,36
Whole tibia	Density (mm Al equiv/mm^3^)	1.60	0.01	1.61	0.01	0.412	1,36
	% cortical bone	56.84	2.33	50.84	2.30	0.075	1,36
Tibia cortical							
Tibia cortical	Density (mmAl equiv/mm^3^)	0.018	0.001	0.022	0.001	0.024	1,36
Tibia cortical	MinCO3 1415	0.209	0.003	0.170	0.003	< 0.001	1,36
Tibia cortical	CI 1030/1020	0.584	0.004	0.633	0.007	< 0.001	1,36
Tibia cortical	PO_4_/Amide I	3.97	0.11	6.40	0.12	< 0.001	1,36
Tibia cortical	LNK 1660/1690	2.54	0.12	4.17	0.19	< 0.001	1,36
Tibia cortical	AngSpread 002	54.9	0.91	49.4	0.66	0.004	1,35
Tibia cortical	FWHM002	0.45	0.01	0.46	0.017	0.842	1,36
Tibia medullary	Density (mmAl equiv/mm^3^)	0.025	0.003	0.024	0.002	0.743	1,36
Tibia medullary	MinCO3 1415	0.18	0.03	0.16	0.02	0.576	1,36
Tibia medullary	CI 1030/1020	0.77	0.022	0.73	0.02	0.149	1,36
Tibia medullary	PO_4_/Amide I	1.17	0.09	1.25	0.10	0.585	1,36
Tibia medullary	LNK 1660/1690	3.20	0.09	3.20	0.07	0.998	1,36
Tibia medullary	FWHM002	0.58	0.03	0.56	0.02	0.425	1,35

#### Differences in the CBS sequence between the two genotypes and amino acid coding consequences

Using the NGS data, it was possible to build on the *CBS* predicted sequence, ENSGALT00000026110, and to determine the sequence of the two genotypes. This data defined a slightly longer 3′ end in the transcript than in the database and suggested a transcription start site that corresponded to the second predicted exon.

The sequence was submitted to ENSEMBL (EMBL Accession number LR588428). Using this information, the coding sequence was predicted to have 524 amino-acids. There were nine SNPs in the cDNA that formed two haplotypes. These were previously described and can be found in dbSNP as rs317751309, rs736880045, rs315389726, rs314405634, rs314750178, rs316841302, rs15382187, rs317686139 and are determined in the sequence LR588428 to be synonymous. The exception was an amino-acid altering the SNP at position 111,013,071 (galGal6) and at position 1579 in the sequence LR588428, which is annotated as rs316554658. This alters amino-acid 498 in the predicted CBS protein from a lysine (K) AAA to a glutamine (Q) CAA. These are respectively a charged and an uncharged but polar amino-acid. The lysine version is associated with the high bone strength genotype. The SNP rs316554658 was genotyped using RFLP as described above.

#### CBS enzyme activity assay in livers of embryos expressing protein from allelic variants

The Vmax and Km values were derived from samples taken from individual embryos that were homozygous for the amino-acid difference in the CBS sequence. We found no statistical difference in the activity of CBS allozymes measured in the liver of embryos homozygous for the amino-acid 498 coding difference. The kinetic parameters for the two allozymes were; lysine form Km, 0.51 ± 0.10, n = 6; glutamine form Km, 0.62 ± 0.13, n = 9 and lysine form Vmax, 107.9 ± 44.0, n = 6; glutamine form Vmax, 157.4 ± 52.4, n = 9.

## Discussion

Our results provide strong evidence that differences in the sequence surrounding the *CBS* gene are responsible for the observed phenotypic effects of the QTL on bone quality that was previously observed [16] and fine-mapped in the current study. Specifically, the difference in expression between individual hens that carry different combinations of the alleles is persuasive. There are a number of reasons, besides the obvious large difference in expression, which lead us to this conclusion. Cystathione beta synthase has a key role in the methionine cycle as part of the one-carbon metabolic cycle in the production of sulphur-containing-amino-acids and is involved through its substrate in bone health [41]. CBS acts on its substrate, homocysteine, to regulate the conservation of methionine or the synthesis of cysteine via the trans–sulfuration pathway. The *CBS* gene has been reported to be highly expressed in embryonic and post-natal bone [42] and there is considerable evidence that high homocysteine levels, may affect collagen cross-linking and hence osteoporosis e.g. [43–45]. However, there is limited evidence to support any specific mechanism. Early studies suggested that high levels of homocysteine resulted in higher solubility of collagen from a small number of affected individuals [46] and it is suggested that the mechanism may be a decrease in the activity of lysyl oxidase, which catalyses the crosslinking of collagen observed in vivo in chicks [47]. However the effect was not observed in vitro, so the inhibition was not assumed to be direct [47].

There were other genes for which the fold change in expression between the genotypes was much less than that for *CBS*, three at the same locus as *CBS* on chromosome 1 and one on chromosome 4. *Ribosomal RNA processing 1B* (*RRP1B*) is involved in ribosomal production but the literature focuses on its effects on extracellular matrix gene expression, tumor growth, and metastasis of cancer cells [48]. There are no reports of effects on bone although the extracellular matrix can of course include collagens. *Salt inducible kinase 1* (*SIK1*) plays a role in conserved signal transduction pathways and may be part of a mechanism that maintains sodium balance in cells [49]. There is one report that suggests that it may play a role in osteoclast differentiation [50]. *Phosphatidylinositol glycan anchor biosynthesis class P* (*PIGP*) is a component involved in the catalysis of glycolysis of proteins. We found no reports of a role in bone, but it is involved in blood cell glycolysis. The gene that was not at the locus on chromosome 1 was *DExD/H*-*box helicase 60* (*DDX60*) and is principally recognised as an RNA binding molecule with anti-viral properties, but it has also been mentioned as a potential candidate for osteoporosis in a study on human monocytes [51].

Since we started with the discovery of a QTL in an F2 population, we fine mapped the locus by returning to the founder population from which the high and low bone strength hens used in the cross were divergently selected. This allowed us to benefit from access to more recombinants. The same denser marker set was also used in the original F2 population and both approaches, as expected, identified a similar region.

The SNPs at the QTL were highly significantly associated with tibia strength, with an additive effect of ~ 8 Newton in breaking strength. This represents a large effect given a population mean for breaking strength of ~ 200 Newton. As expected for a genuine QTL, the addition of more markers in the F2 population improved the confidence of the result and therefore narrowed down the region. The QTL seems to be located on chromosome 1 between 104.1 and 110.4 Mb on the galGal6 assembly, whereas the flanking markers from the F2 population put the location at galGal6, Chr 1: 107.0-113.1 for bone index and the bone index component, tibiotarsus breaking strength, although the estimated QTL peak position for the humerus breaking strength was galGal6, Chr 1: 90.2 Mb. The positions for the bone index and tibiotarsus QTL were strongly supported by the evidence from the NGS expression data from mid-shaft tibia from individuals with the Ost112522587/Ost106225194; *AA*/*AA* vs. *GG*/*GG* genotypes. Between the two genotypes, we observed a group of genes with differences in expression that clustered around the gene with the largest expression difference, *CBS* on chromosome 1 (galGal6, Chr 1: 111,011,730-111,028,603).

Expression data from heterozygotes for the low and high bone strength alleles showed allelic imbalance in the expression of *CBS*. This clearly indicated that the difference in expression was due to a cis-acting effect and not a trans-acting effect. In other words, it was unlikely that there was any involvement of a transcription factor transcribed from the region, acting on expression, as this would affect both alleles. In a complementary paper, we have identified a region of six tandem repeats in the promoter region of the *CBS* gene, which resulted in differences in the level of methylation and transcriptional activity, and which segregates with the alternative genotypes [32]. This may explain the differences in expression.

However, applying Occam’s razor, the simplest possibility for the observed differences in bone strength between the genotypes might be the difference in the predicted amino-acid sequence of the *CBS* gene at position 498 from a lysine (K) to a glutamine (Q). Differences in expression could be the result of differences in feedback mechanisms if the enzyme activity was more or less active than the ‘wild type’ gene and protein. However, the results from the allelic imbalance study does not support the hypothesis that the observed effects were due to differences in feedback because of a faulty copy of the CBS enzyme. If a faulty copy of CBS was present, we would expect both copies to be equally affected by any feedback in a heterozygous individual and this was not the case. Of course, it could be that a combination of differences in the enzyme and a site in the promoter or enhancer through which the feedback mechanism works could be linked, but this seems less likely. Finally and more directly, we did not observe a difference in the activity of the two allozymes when they were tested. The chicken and the turkey genomes predict a glutamine (Q) at position 498 in the CBS protein, and glutamine has a non-charged polar side chain. Lysine (K), which has a charged side chain seems universal in predictions from other bird genomes. Lysine is also present in the chicken at this position when the glutamine codon is not present, as we have seen in this study. Reptiles feature glutamine (Q) or aspartic acid (D) at this position in the CBS protein; these amino-acids possess a charged side chain with a negative charge. In mammals, it seems that threonine (T), another non-charged polar side chain amino-acid, is almost universal. Therefore, there is no indication that the charge at the position is conserved or that the difference in charge at this position might lead to a large effect on the enzyme’s activity. The results of the assays of enzyme activity in our study confirmed this with neither Vmax nor Km differing between the forms.

The level of plasma homocysteine, which is the substrate for the CBS enzyme, differed significantly between the genotypes at 48 and 70 weeks of age, with plasma concentrations of homocysteine being about 2 µMol/L higher for *AA* hens than for *GG* hens at both ages. The difference is relatively small (~ 10%), in comparison to the difference in expression of the gene that is about nine-fold. In a study on human patients, in which groups from the extreme ends of the observed range were constituted, the means for plasma homocysteine were 7 and 28 μMol/L [45]. In our population, the maximum and minimum levels of homocysteine observed were 6 and 26 μMol/L. If we used a similar approach to that of the human study, the extreme groups would have means of 12 and 23 μMol/L, respectively. Therefore, the distribution of homocysteine values in the plasma from a normal population of chickens varies to a similar extent to that observed in humans. In humans, the correlations observed between plasma homocysteine and collagen cross-links [45] were across a much larger range of plasma homocysteine concentrations than what we observed in this study.

Therefore, contrary to what was expected, we observed both higher gene expression and higher homocysteine levels in the plasma of carriers of the *AA* allele associated with lower bone strength. At least, this is consistent with the observation that higher plasma homocysteine is associated with poorer bone quality. Since we have established that there is no large difference in the activity of the enzyme between genotypes, we could expect higher gene expression to be correlated with increased protein activity and a potential reduction in the substrate homocysteine [35]. Certainly, the presence of inactivating mutations in the *CBS* gene results in hyperhomocysteinemia [52]. It is also stated that mutations in CBS result in only mild increases in plasma homocysteine but these are more evident after a methionine load, with more marked effects being observed in defects of the re-methylation pathway [53].

Across the different analyses performed in this paper, we see a consistent effect of the *GG* genotype being associated with greater bone strength. The exceptions were the analyses involving small numbers of hens, but this is almost certainly an effect of power. For example, for the samples used for NGS, the difference in cortical density tended to show a denser structure only for the *GG* genotype although the medullary bone was significantly denser in individuals with the *GG* genotype. However, there was a very clear effect on gene expression.

Similarly, in the samples for which we examined the physicochemical properties of the bone between genotypes, we did not observe a significant effect on the bone mechanical properties but there were clear effects on the bone mineral chemistry and structure properties. Specifically, there was a higher degree of mineralization and higher degree of crystal orientation in cortical bone in individuals with the *GG* genotype, which was accompanied by lower MinCO_3_ 1415 in the tibia cortical bone. All these characteristics are typical of more mature bone, which has a higher degree of mineralization. This could be caused by a decreased turnover rate that slows down the renewal of bone tissue [37, 54]. There was also more crosslinking of collagen in the tibial cortical bone (LNK 1660/1690), which is characteristic of older bone tissue. This suggests that the bone from the stronger genotype was associated with greater mineralisation, which is consistent with its higher density, the bone is also more mature, possibly with a higher degree of cross-links in the collagen which is not favoured by high homocysteine levels and therefore agrees with the observed lower plasma homocysteine [45]. Mineralization of the bone organic matrix occurs via the oriented nucleation of apatite crystals within the collagen fibre gaps as well as in the outer surface of collagen fibrils [55]. Any change in collagen structure caused by different levels of homocysteine could impact the mineralization of bone as in osteomalicia or osteogenesis imperfecta, which are caused by an alteration of collagen structure and produce abnormal mineralization of the organic matrix [38, 56, 57].

These differences in bone properties are in contrast to the observations made between the divergently selected lines that were used to create the original F2, which were derived from the same line [37]. Between these lines there were no effects, although exercise did result in changes to the physico-chemical attributes of the bone [37], many of which are similar to those observed in this study, including the increased cross-linking. However, in that study the bones were sampled from hens that probably differ at many genetic loci as they were selected on the trait, not on a specific genotype. Whereas, in the samples examined in our study for the physico-chemical attributes of the bone, the hens were selected on a single genetic locus. Although the locus explained a relatively large effect, this was still small compared to the difference between the selected lines that gave rise to the F2 individuals that were initially used to discover the QTL [8]. Indeed this exemplifies the problems of studying the individual locus underlying variation in a quantitative trait. Even if the locus effect is relatively large, it is still likely to reach a magnitude that, for many measurements related to the trait, would require extremely large numbers of animals to resolve at a physiological or biochemical level how exactly the genetic locus exerts its effect.

There are a number of independent studies that have identified genetic loci for bone quality by using mainly crosses between fast and slower growing chicken strains with a risk of confounding effects of body weight; these studies include QTL that show relatively wide confidence limits, some which coincide with the QTL studied in this manuscript [58]. Our study and other previously published ones have located a number of loci across the genome, which are potential candidates for bone quality, some of which may be relevant to layers, often featuring bone density or mineral content [12, 13, 58–60]. Although some report QTL on chromosome 1, these do not appear to coincide with the QTL that we fine-mapped in this study [61, 62]. Using a genome-wide association approach in one of the populations in this study, loci with a larger effect than the *CBS* loci have been observed, which suggests we can make progress in finding the underlying mechanism for these loci if large enough samples can be assembled [63].

## Conclusions

We have confirmed and fine-mapped a genetic locus that affects the mechanical and physico-chemical properties of bone in laying hens. The physico-chemical properties of the bones from the two genotypes suggest that greater mineralisation and maturity of collagen cross-linking may be responsible for the improved quality of bone. We have identified a gene that encodes an enzyme, i.e. the *CBS* gene, which shows significantly different levels of expression between genotypes at the QTL. Adjacent genes to *CBS* are also differentially expressed, which suggests that a *cis*-acting enhancer operates at the locus. The genotype associated with higher expression of the *CBS* gene and poorer bone quality shows a small but significantly increased expression in the enzyme’s substrate, homocysteine. A number of studies have shown effects of raised plasma homocysteine in relation to poor bone quality but the effects reported here are small by comparison to those studied in humans. Although there are differences in the CBS protein sequence at one amino-acid, we cannot detect a difference in enzyme activity that could explain the observed phenotype in bone quality, and the difference in expression of the gene and the effect on the substrate concentration are not consistent. Therefore, although we have confirmed and extended the observations on this locus and have revealed the underlying cause of the phenotype, which is a localised region of gene expression differences, we cannot say that we have proven that *CBS* is the gene responsible. It may be necessary to look at other genes in the region to answer the question of which gene or genes make a difference to bone quality. From a practical point of view, in the meantime, markers at this locus can be used to improve bone quality and nutritional interventions to modify homocysteine levels and thus improve bone quality are possible.

## Supplementary information

**Additional file 1: Table S1.** Genetic map for the resolution of the QTL for bone quality in the F2 population. Marker names and distance between markers in cM.

**Additional file 2: Table S2.** SNPs used in this study with their position on GalGal6. SNPs were derived from dbEST, sequencing and dbSNP.

**Additional file 3.** Full details on the methods used in the paper relating to RNAseq, liquid chromatography and mass spectrophotometry and measurement of physicochemical characteristics of bone [25–27, 37–39, 54, 64–67].

**Additional file 4:** Mass Spectrophotometer transitions. The eluent from LC was passed onto the electrospray source of an amaZon ETD Ion Trap operated in MRM mode with the following transitions. The concentrations of components in samples were calculated by comparison with external calibration curves of authentic compounds.

## Data Availability

The NGS data were submitted to the European nucleotide archive (study accession PRJEB6782) and used in the annotation of the chicken genome. Sequence data related to *CBS* were submitted to ENSEMBL (EMBL Accession LR588428). The pedigree data used during the current study for population 2 are not publicly available, due to data restrictions regarding the pedigree information from Lohmann Tierzucht. All other data pertaining to the studies along with the supplementary tables have been archived with the following accessible doi number 10.7488/ds/2619.
