# Supplementary material for: An eQTL in the cystathionine beta synthase gene is linked to osteoporosis in laying hens
Source: Genet Sel Evol. 2020 Feb 24;52:13. doi: 10.1186/s12711-020-00532-y (PMC7038551; doi:10.1186/s12711-020-00532-y)
Supplement: Supplementary file 1 — Additional file 1: Table S1. Genetic map for the resolution of the QTL for bone quality in the F2 population. Marker names and distance between markers in cM. [file 12711_2020_532_MOESM1_ESM.docx]

**Additional file 1**

Format: .docx

Title: Table S1, Genetic map for the resolution of the QTL for bone quality in the F2 population.

Description: Marker names and distance between them in cM.

| **Marker or SNP ID** | **Distance in cM on chromosome between markers** |
| --- | --- |
| ROS0008 | 3.4 |
| ADL0160 | 1.103087 |
| rs14791127 | 0.546126 |
| rs14791031 | 0.789083 |
| rs13826562 | 0.288597 |
| rs13826521 | 2.764638 |
| rs13826119 | 0.131783 |
| rs16692189 | 0.301683 |
| rs15189059 | 0.538662 |
| rs13825877 | 0.242872 |
| rs15188694 | 0.693738 |
| rs13825609 | 1.541966 |
| rs14789971 | 1.29313 |
| rs14797198 | 0.936505 |
| rs15203129 | 0.907914 |
| rs13833945 | 0.289335 |
| rs13715018 | 0.465672 |
| rs14796746 | 0.33073 |
| rs14796720 | 0.16506 |
| MCW0036 | 0.758136 |
| rs13833397 | 0.4297 |
| rs14796399 | 6.133426 |
| rs14792587 | 2.160409 |
| rs14792295 | 0.503671 |
| rs13828303 | 0.298135 |
| rs14792100 | 0.259724 |
| rs14792064 | 0.391034 |
| rs13827931 | 0.183589 |
| rs14791775 | 0.151594 |
| HUJ0004 | 1.404483 |
| rs13827618 | 0.514605 |
| rs14791532 | 1.580912 |
| GCT0006 | 1.951948 |
| rs14797607 | 3.202839 |
| rs14797963 | 2.123832 |
| rs15205802 | 1.233088 |
| rs14692821 | 1.174232 |
| rs14798314 | 0.940216 |
| rs13836249_2 | 0.853112 |
| rs14798473 | 0.68751 |
| rs14798575 | 1.857641 |
| rs15206987 | 0.375583 |
| LEI0194 | 0.348732 |
| rs14798895 | 6.650042 |
| rs13837741 | 4.171322 |
| rs13746405 | 16.42582 |
| rs14800758 | 23.80408 |
| MCW0011 | 0.72482 |
| rs13841828 | 1.550757 |
| rs13747686 | 2.991372 |
| rs13842237 | 2.165755 |
| rs13747869 | 2.601112 |
| rs15217520 | 2.088272 |
| rs14803499 | 8.708962 |
| rs15218822 | 1.696322 |
| rs14804228 | 0.877353 |
| rs14804265 | 0.795277 |
| MCW0106 | 1.028701 |
| rs13843741 | 2.140505 |
| rs13843952 | 11.6278 |
| rs13748804 | 3.86432 |
| rs14805816 | 2.32568 |
| rs13749003 | 5.895964 |
| rs14806488 | 1.133669 |
| rs13845970 | 4.781579 |
| rs15225888 | 2.339785 |
| rs13749468 | 1.199369 |
| rs14807010 | 0.894394 |
| rs14807068 | 4.788703 |
| rs14807281 | 8.059801 |
| rs13847411 | 0.916312 |
| rs13847439 | 0.594143 |
| rs13847466 | 10.31111 |
| rs14808446 | 1.035099 |
| rs13750139 | 0.622809 |
| rs15229887 | 2.740254 |
| GCT0015 | 2.039081 |
| rs14809238 | 3.891331 |
| rs13849993 | 1.025285 |
| rs14810075 | 1.668085 |
| rs15233810 | 1.343058 |
| rs15234281 | 0.23316 |
| ADL0019 | 10.11481 |
| rs15236615 | 0.485189 |
| ADL0307 | 22.2 |
| LEI0146 | 28.1 |
| MCW0112 | 1 |
| ADL0150 | 0 |
| LEI0101 | 43.4 |
| ADL0268 | 16.5 |
| MCW0061 | 5.7 |
| ADL0020 | 10.3 |
| LEI0108 | 5.5 |
| LEI0217 | 2 |
| LEI0128 | 1.985882 |
| Ost92365348 | 0.314118 |
| GCT0051 | 1.6 |
| MCW0109 | 7.402838 |
| Ost99883015 | 0.597162 |
| ROS0081 | 10.7 |
| MCW0208 | 1.6 |
| GCT0013 | 0.254601 |
| Ost101413556 | 0.345399 |
| ADL0037 | 1.5 |
| ADL0148 | 8.739156 |
| Ost109151638-2006 | 0.000313 |
| Ost109151769-2006 | 0.538561 |
| Ost106225194 | 0.562327 |
| Ost106460620 | 0.86746 |
| Ost106823022 | 0.279184 |
| Ost106940170 | 0.86 |
| Ost110455031-2006 | 0.21 |
| Ost107389494 | 0.440765 |
| LEI0198 | 0.082976 |
| Ost107766125 | 0.106717 |
| Ost108015093 | 0.510441 |
| Ost112374543-2006 | 0.062924 |
| Ost112522587-2006 | 0.453615 |
| Ost110373245 | 0.064005 |
| Ost113740421-2006 | 0.001193 |
| Ost113743229-2006 | 0.384928 |
| Ost114648871-2006 | 0.409139 |
| Ost115611476-2006 | 0.002579 |
| Ost115617544-2006 | 0.016277 |
| Ost115655839-2006 | 0.087453 |
| Ost115861595-2006 | 0.000302 |
| Ost115862305-2006 | 0.001265 |
| Ost115865281-2006 | 0.000418 |
| Ost115866264-2006 | 1.41577 |
| LEI0139 | 2.86388 |
| Ost121757410-2006 | 2.901846 |
| Ost121237272 | 2.354031 |
| Ost126455926-2006 | 7.348162 |
| Ost129219873 | 1.358232 |
| Ost134239048-2006 | 0.000211 |
| Ost134239237-2006 | 2.573637 |
| LEI0106 | 28.5 |
| LEI0084 | 21.8 |
