# Supplementary material for: An eQTL in the cystathionine beta synthase gene is linked to osteoporosis in laying hens
Source: Genet Sel Evol. 2020 Feb 24;52:13. doi: 10.1186/s12711-020-00532-y (PMC7038551; doi:10.1186/s12711-020-00532-y)
Supplement: Supplementary file 2 — Additional file 2: Table S2. SNPs used in this study with their position on GalGal6. SNPs were derived from dbEST, sequencing and dbSNP. [file 12711_2020_532_MOESM2_ESM.docx]

**Additional file 2**

File Format: .docx

Title: Table S2, SNPs used in this study with their position on GalGal6.

Description: SNPs were derived from dbEST, sequencing and dbSNP.

| **Marker name** | **GalGal6 position** | |  |
| --- | --- | --- | --- |
| rs14791127 | 5631976 |  | |
| rs14791031 | 5900662 |  | |
| rs13826562 | 6299946 |  | |
| rs13826521 | 6418519 |  | |
| rs13826119 | 7683739 |  | |
| rs16692189 | 7755756 |  | |
| rs15189059 | 7894847 |  | |
| rs13825877 | 8110330 |  | |
| rs15188694 | 8225306 |  | |
| rs13825609 | 8556785 |  | |
| rs14789971 | 9324539 |  | |
| rs14797198 | 9647204 |  | |
| rs15203129 | 10094764 |  | |
| rs13833945 | 10533636 |  | |
| rs13715018 | 10671460 |  | |
| rs14796746 | 10898536 |  | |
| rs14796720 | 11049581 |  | |
| rs13833397 | 11483070 |  | |
| rs14796399 | 11682171 |  | |
| rs14792587 | 15028358 |  | |
| rs14792295 | 16181995 |  | |
| rs13828303 | 16419237 |  | |
| rs14792100 | 16564974 |  | |
| rs14792064 | 16685623 |  | |
| rs13827931 | 16893991 |  | |
| rs14791775 | 16993653 |  | |
| rs13827618 | 17612771 |  | |
| rs14791532 | 17825917 |  | |
| rs14797607 | 19065411 |  | |
| rs14797963 | 19969344 |  | |
| rs15205802 | 20538674 |  | |
| rs14692821 | 20838801 |  | |
| rs14798314 | 21120113 |  | |
| rs14798473 | 21580304 |  | |
| rs14798575 | 21758951 |  | |
| rs15206987 | 22206030 |  | |
| rs14798895 | 22347555 |  | |
| rs13837741 | 22914790 |  | |
| rs13746405 | 23271715 |  | |
| rs14800758 | 24683817 |  | |
| rs13841828 | 26800917 |  | |
| rs13747686 | 26935365 |  | |
| rs13842237 | 27194387 |  | |
| rs13747869 | 27382933 |  | |
| rs15217520 | 27608924 |  | |
| rs14803499 | 27790129 |  | |
| rs15218822 | 28573291 |  | |
| rs14804228 | 28719353 |  | |
| rs14804265 | 28796156 |  | |
| rs13843741 | 28945050 |  | |
| rs13843952 | 29109239 |  | |
| rs13748804 | 29998054 |  | |
| rs14805816 | 30293112 |  | |
| rs13749003 | 30470253 |  | |
| rs14806488 | 30919580 |  | |
| rs13845970 | 31006943 |  | |
| rs15225888 | 31371111 |  | |
| rs13749468 | 31552169 |  | |
| rs14807010 | 31644102 |  | |
| rs14807068 | 31712282 |  | |
| rs14807281 | 32078013 |  | |
| rs13847411 | 32687362 |  | |
| rs13847439 | 32738439 |  | |
| rs13847466 | 32783843 |  | |
| rs14808446 | 33551085 |  | |
| rs13750139 | 33629615 |  | |
| rs15229887 | 33677011 |  | |
| rs14809238 | 34245240 |  | |
| rs13849993 | 34922353 |  | |
| rs14810075 | 35100848 |  | |
| rs15233810 | 35391935 |  | |
| rs15234281 | 35623806 |  | |
| rs15236615 | 36585216 |  | |
| Ost92365348 | 90332842 |  | |
| Ost99883015 | 97883872 |  | |
| Ost101413556 | 99395470 |  | |
| Ost106225194 | 104084042 |  | |
| Ost106460620 | 104314846 |  | |
| Ost106823022 | 104678816 |  | |
| Ost106940170 | 104834987 |  | |
| Ost107389494 | 105280127 |  | |
| Ost107766125 | 105655660 |  | |
| Ost108015093 | 105901013 |  | |
| Ost109130111 | 107015240 |  | |
| Ost109140396 | 107025525 |  | |
| Ost109151638 | 107036767 |  | |
| Ost109151769 | 107036898 |  | |
| Ost110373245 | 108261209 |  | |
| Ost110453343 | 108340708 |  | |
| Ost110455031 | 108342396 |  | |
| Ost112372739 | 110244688 |  | |
| Ost112374543 | 110246492 |  | |
| Ost112377406 | 110249355 |  | |
| Ost112383168 | 110255116 |  | |
| Ost112388430 | 110260378 |  | |
| Ost112520125 | 110391255 |  | |
| Ost112522587 | 110393717 |  | |
| Ost113740421 | 111546531 |  | |
| Ost113743229 | 111549338 |  | |
| Ost114648871 | 112392632 |  | |
| Ost115611476 | 113329344 |  | |
| Ost115617544 | 113335412 |  | |
| Ost115655777 | 113373094 |  | |
| Ost115655839 | 113373156 |  | |
| Ost115861595 | 113578876 |  | |
| Ost115862305 | 113579586 |  | |
| Ost115865281 | 113582562 |  | |
| Ost115866264 | 113583545 |  | |
| Ost119070424 | 116194725 |  | |
| Ost119052645 | 116212504 |  | |
| Ost121237272 | 119214984 |  | |
| Ost121554877 | 119531164 |  | |
| Ost121562647 | 119538934 |  | |
| Ost121757410 | 119732454 |  | |
| Ost122391295 | 120362419 |  | |
| Ost122391488 | 120362612 |  | |
| Ost122391626 | 120362750 |  | |
| Ost122391682 | 120362806 |  | |
| Ost122391709 | 120362833 |  | |
| Ost126468119 | 124851100 |  | |
| Ost126455926 | 124863293 |  | |
| Ost129219873 | 126721130 |  | |
| Ost134239048 | 131424865 |  | |
| Ost134239237 | 131425054 |  | |
