# Supplementary material for: An eQTL in the cystathionine beta synthase gene is linked to osteoporosis in laying hens
Source: Genet Sel Evol. 2020 Feb 24;52:13. doi: 10.1186/s12711-020-00532-y (PMC7038551; doi:10.1186/s12711-020-00532-y)
Supplement: Supplementary file 3 — Additional file 3. Full details on the methods used in the paper relating to RNAseq, liquid chromatography and mass spectrophotometry and measurement of physicochemical characteristics of bone [25–27, 37–39, 54, 64–67]. [file 12711_2020_532_MOESM3_ESM.docx]

**Additional file 3**

Format: .docx

Title: Full details for methods, includes references [25-27, 37-39, 54, 64-67].

Description: Full details on the methods used in the paper relating to RNAseq, Liquid chromatography and mass spectrophotometry and measurement of physicochemical characteristics of bone.

**RNA seq**

From Population 4, samples were selected by choosing among hens for which the egg was in the shell gland to reduce any effect of the egg calcification cycle. Half the samples were homozygous for low bone breaking strength genotype (n = 8) and the other half (n = 8) were homozygous for the high bone breaking strength genotype. Samples were prepared for mRNA sequencing using 1 µg of total RNA starting material following the Illumina Truseq RNA sequencing protocol. The libraries were quality-checked on an Agilent DNA 1000 bioanalyzer (Agilent Technologies, South Queensferry, UK) and then clustered onto a paired-end flow cell using the Illumina v3 cluster generation kit at a 8 pM concentration. One-hundred cycle paired-ended sequencing was carried out on the HiSeq 2000 using Illumina v2 Sequencing by Synthesis kits (Illumina, Little Chesterford, UK). The Illumina HiSeq 2000 platform (Edinburgh Genomics) generated between 40 to 60 million RNAseq tags per sample (819 million in total), each 100 nucleotides long – resulting in 81.85 Gb of data. The raw reads were aligned to the chicken genome (release 69) using bwa version 0.6.2 and samtools version 0.1.18 with default parameters. Results from the *bam files were aligned to the genome. The reads hitting each gene were counted for each sample (HTSeq version 0.5.3p9) and were counted only once, even if both ends mapped. Differential expression of genes or tags was assessed using edgeR [25], a package in the bioconductor suite [26] implemented in R [27]. Data from low expressing tags were removed and the data normalised based on its overall variance and the variance of individual tags. The likelihood that the expression of genes differed between the genotypes was estimated using a general linear model in the edgeR package version 3.6.8.

**LC-MS method**

Amino acids and their derivatives were separated from either cell culture medium or plasma eluate using an Ultimate HPLC system on a zic-hilic column (Merck Millipore, 2.1mm id x 15 cm long) applying a flow rate of 0.2 mL/min of solvents. A solvent system of 10 mM ammonium formate and 0.15% formic acid (solvent A) and acetonitrile (solvent B) was used as mobile phase. A gradient profile starting at 5% A for 2 min, increasing to 35% of solvent A by 7 min was applied, followed by an increase to 95% of A within 1 min. This was followed by a 2-minute hold at 95% of A, returning to 5% of A and a final hold to re-equilibrate the column for 4 minutes.

The eluent from LC was passed onto the electrospray source of an amaZon ETD Ion Trap (Bruker Daltonics GmbH, Bremen, Germany) operated in MRM mode with the following transitions. [see Additional file 4 Table S3]. The mass spectra were obtained in the ultrascan mode in the m/z scan range 70–300, with an ion charge control (ICC) target setting 100,000 and a maximum accumulation time 100 ms. Data analysis 4.2 and Quant analysis (Bruker) software were used to generate Extracted Ion Chromatograms (EIC) of [M+H] + ions of each analyte with an m/z window for EIC ± 0.3 Da. The concentration of components in the samples were calculated by comparison with external calibration curves of authentic standards. Standards were prepared in the sample solution but cystathionine concentration varied between 7 and 450 µmol/L.

**Bone material properties**

**Bone chemistry and microstructure**

***Bone samples:*** Tibia bones were stored in a freezer at -20 ºC until analysed for bone physicochemical material specific properties (e.g., bone microstructure, chemical composition of the cortical and medullary bone, mineral crystallinity and crystal orientation, and collagen maturity) using, infrared spectroscopy and X-ray diffraction techniques as described below.

***Infrared spectrometry:*** The chemical composition of bone tissues (cortical and medullary bone) were analyzed by infrared spectroscopy as previously described [37]. The relative amounts of water, proteins (collagen), lipids, phosphate and carbonate in the bone samples were determined from the peak area of the absorption bands associated with the characteristic molecular groups of each component [38, 39]. Specifically: (1) the relative amount of mineral to organic matrix (PO4/Amide I) was determined as the ratio of the main phosphate (v1, v3 PO4; 900 - 1200 cm-1) to Amide I (1590-1710 cm-1) band area ratio; (2) total carbonate relative to mineral content (MinCO3 1415) was determined as the ratio of the main carbonate band (v3 CO3; 1390-1440 cm-1) to the main phosphate band (900 - 1200 cm-1) area ratio, which decreases as bone mineral matures [64, 65]; (3) the mineral crystallinity index (CI 1030/1020) was determined as the ratio between phosphate sub-bands 1030 and 1020 cm-1 areas, which increases as the mineral crystal size and perfection increases [54]; (4) the amount of collagen cross-links was determined as the area ratio between amide I sub-bands at 1660 and 1690 cm-1 (LNK 1660/1690), which increases with collagen maturity [54, 66].

***X-ray diffraction:*** Tibiae cortical bone (about 1x1 cm) cuts from the diaphysis were analyzed in transmission mode with a single crystal diffractometer equipped with an area detector (D8 SMART APEX from Bruker) and Mo radiation (50 KV and 30 mA; 0.5 mm collimator). A quantitative estimation of the degree of orientation of apatite crystals (Angular spread; AS) in the cortical bone was determined from the angular breadth of bands displayed in the intensity profile along the Debye-Scherrer ring associated with the 002 reflection of apatite mineral [37, 67]. The wider the band, the greater the scattering in the orientation of the c-axis of apatite crystals, becoming more randomly distributed. The proportion of oriented apatite crystals in bone mineral (Oriented fraction) was determined as the ratio between the intensity of oriented crystals versus total intensity in 002 Gamma scans [67]. In addition, crystallinity of bone mineral was determined by measuring the full width at half maximum (FWHM) of the main apatite peak (002) displayed in theta-2theta scans determined from powdered samples analyzed with a X-ray powder diffractometer (Panalytical Expert Pro, The Netherlands) with Cu radiation (40 KV and 35 mA).
