# Supplementary material for: An eQTL in the cystathionine beta synthase gene is linked to osteoporosis in laying hens
Source: Genet Sel Evol. 2020 Feb 24;52:13. doi: 10.1186/s12711-020-00532-y (PMC7038551; doi:10.1186/s12711-020-00532-y)
Supplement: Supplementary file 4 — Additional file 4: Mass Spectrophotometer transitions. The eluent from LC was passed onto the electrospray source of an amaZon ETD Ion Trap operated in MRM mode with the following transitions. The concentrations of components in samples were calculated by comparison with external calibration curves of authentic compounds. [file 12711_2020_532_MOESM4_ESM.docx]

**Additional file 4**

Format: .docx

Title: Table S3, Mass Spectrophotometer transitions

Description: The eluent from LC was passed onto the electrospray source of an amaZon ETD Ion Trap operated in MRM mode with the following transitions. The concentrations of components in samples were calculated by comparison with external calibration curves of authentic compounds.

| **Analyte** | **MS2 conditions** | | | **Fragments used for quantification (m/z)** |
| --- | --- | --- | --- | --- |
|  | **Parent ion/m/z** | **Isolation width/m/z** | **Amplitude** |  |
| Serine | 106.1 | 1 | 0.5 | 60.2 |
| S-adenosyl methionine | 298 | 1 | 0.6 | 135.8 |
| Cystathionine | 222.8 | 1 | 0.35 | 133.8 |
| Homocysteine | 135.9 | 1 | 0.5 | 89.9 |
